# Supplementary material for: Clonally diverse CD38+HLA-DR+CD8+ T cells persist during fatal H7N9 disease
Source: Nat Commun. 2018 Feb 26;9:824. doi: 10.1038/s41467-018-03243-7 (PMC5827521; doi:10.1038/s41467-018-03243-7)
Supplement: Supplementary file 1 — Supplementary Information [file 41467_2018_3243_MOESM1_ESM.pdf]

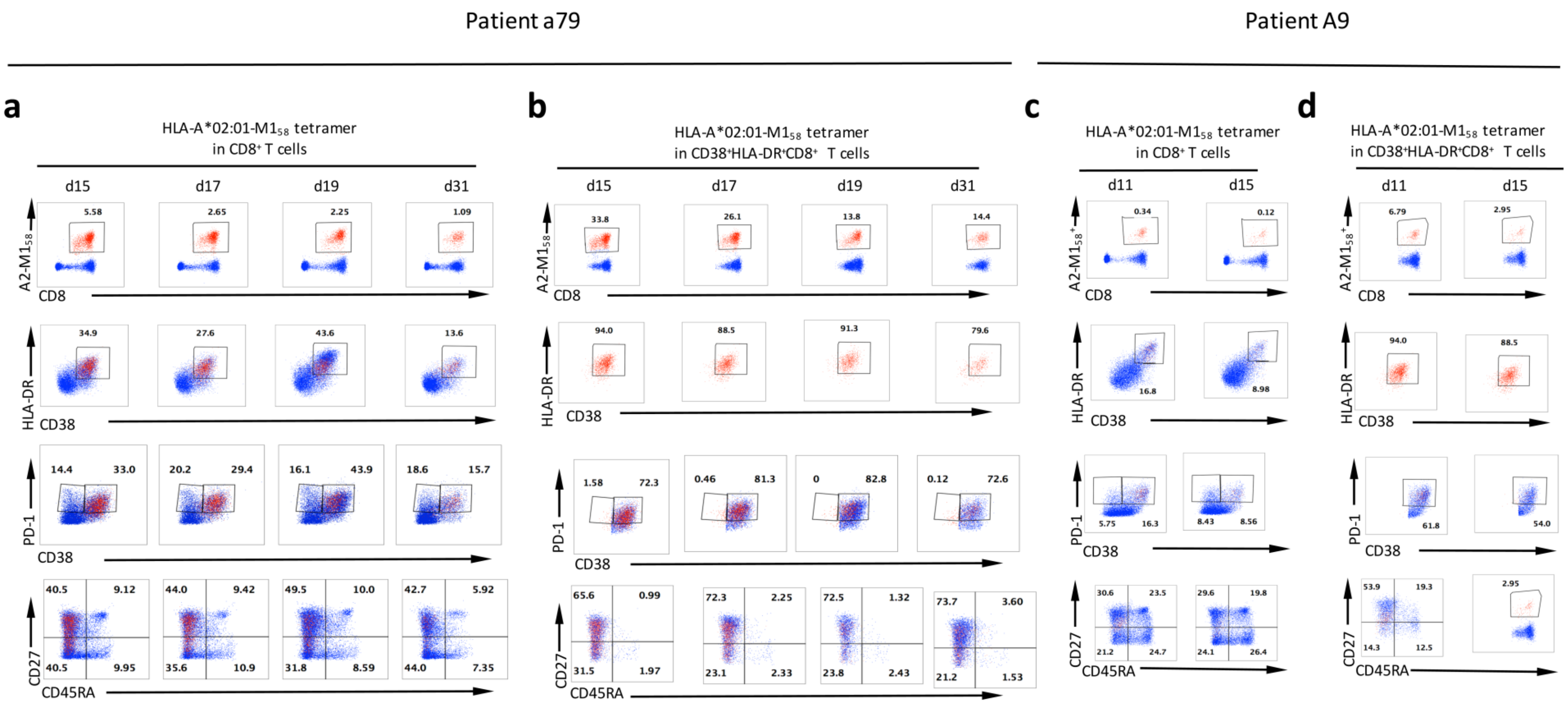

Supplementary Figure 1. Influenza-specific A2-M1$_{58}$-tetramer^{+}CD8^{+} T cells are of the CD38^{+}HLA-DR^{+}PD-1^{+} phenotype during severe H7N9 infection. PBMCs from HLA-A*02:01-positive patients were stained with the A2-M1$_{58}$ tetramer and phenotypic panel described in M&M. Representative FACS plots for A2-M1$_{58}$$^{+}$CD8^{+} T cells (in red) are shown for **(a, b)** a79 patient and **(c, d)** a9 patient, as gated on **(a)** CD3^{+} T cells (in blue) and **(b)** CD8^{+} T cells (in blue). Staining for A2-M1$_{58}$$^{+}$CD8^{+} T cells (in red) and (ii) CD38 and HLA-DR; PD-1 and CD38; and (iv) CD27 and CD45RA is shown. (ii) A2-M1$_{58}$$^{+}$CD8^{+} T cells of CD38^{+}HLA-DR^{+} phenotype (red dots) were also (iii, iv) overlaid on total **(a)** CD8^{+} T cells (blue dots) or **(b)** CD38^{+}HLA-DR^{+}CD8^{+} T cells (blue dots).

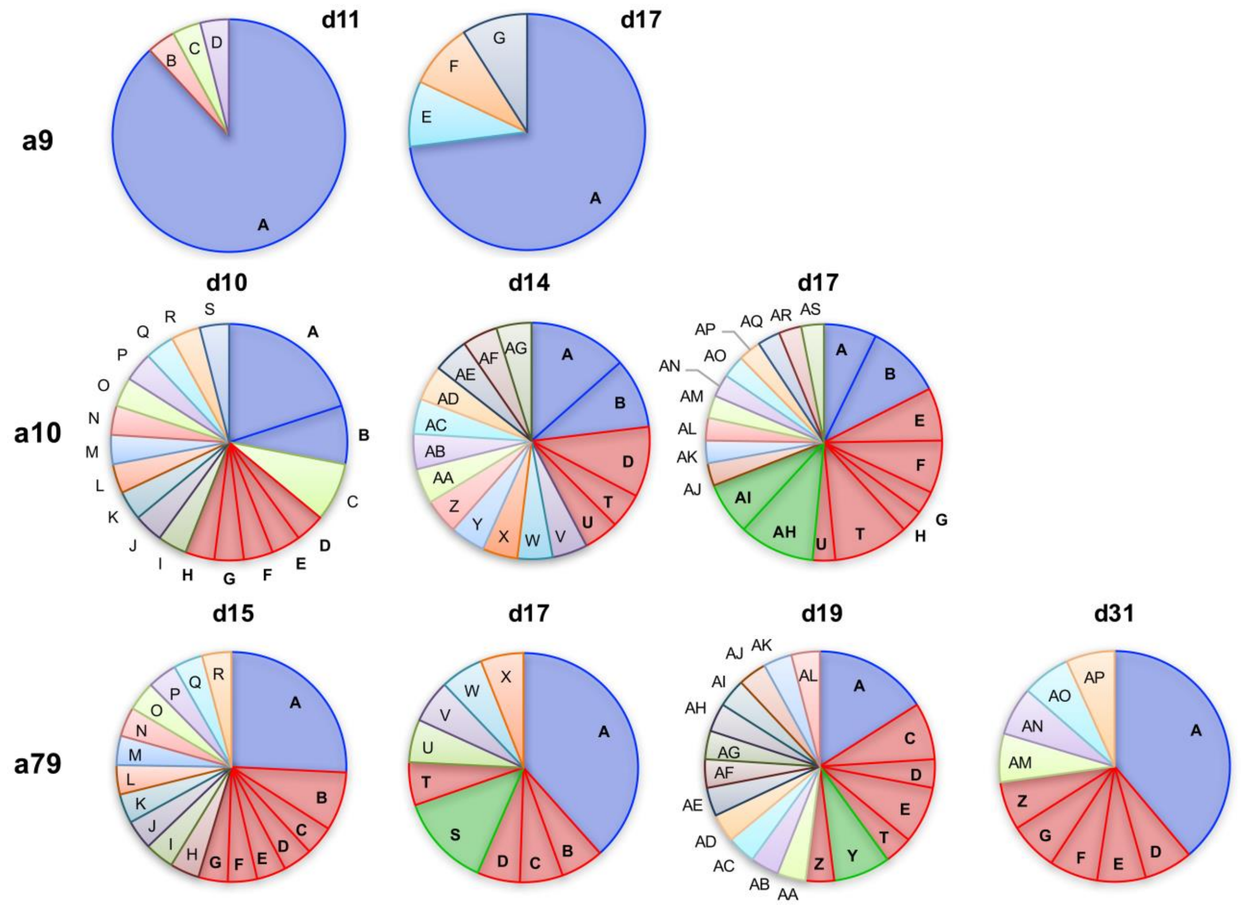

Supplementary Figure 2. **TCRαβ repertoire diversity and stability within A2-M1$_{58}$$^{+}$CD8^{+} T cells in HLA-A*02:01-expressing patients during H7N9 infection.** A2-M1$_{58}$$^{+}$CD8^{+} T cells from three HLA-A*02:01-positive H7N9 patients (a9, a10 and a79) were single cell-sorted for TCRαβ repertoire analysis using a multiplex RT-PCR and sequencing. Populations were gated on viable Dump^{-}tetramer^{+}CD3^{+}CD8^{+} events. Frequency, expressed as pie charts, of paired A2-M1$_{58}$$^{+}$CD8^{+} TCRαβ clonotypes across different time-points after H7N9 infection are shown. Each TCRαβ clonotype is depicted in a different letter code, described in Supplementary Table 1 (a9 patient), Supplementary Table 2 (a10 patient) and Supplementary Table 3 (a79 patient).

TCRαβ repertoire sharing (%)

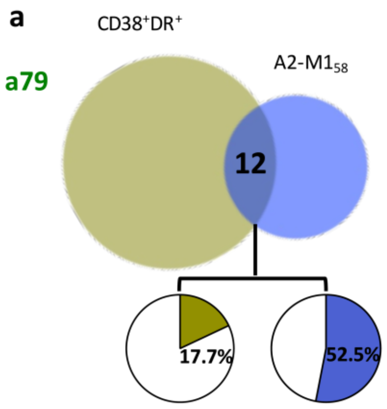

Common TCRαβ clonotypes between CD38^{+}HLA-DR^{+} and A2-M1$_{58}$ CD8^{+} T cell populations.

CDR3α	TRAV	TRAJ	CDR3β	TRBV	TRBJ	CD38+HLA-DR+	A2-M1$_{58}$
DNQGGKL	TRAV5	TRAJ23	SGRSADTQ	TRBV19	TRBJ2-3	1.00	27.50
NYGGSQGNL	TRAV25	TRAJ42	SIRSTGEL	TRBV19	TRBJ2-2	3.33	3.75
AFGSSNTGKL	TRAV27	TRAJ37	SIRSAYEQ	TRBV19	TRBJ2-7	2.22	5.00
SSSNTGKL	TRAV3	TRAJ37	SGRSQETQ	TRBV19	TRBJ2-5	1.11	5.00
AGGGSQGNL	TRAV27	TRAJ42	SGRSTDTQ	TRBV19	TRBJ2-3	1.11	2.50
ADSSSNTGKL	TRAV27	TRAJ37	SIRSAYEQ	TRBV19	TRBJ2-7	1.11	1.25
NYGGSQGNL	TRAV25	TRAJ42	SQRSTDTQ	TRBV19	TRBJ2-3	1.11	1.25
DVSDRGSTLGRL	TRAV22	TRAJ18	SGTSFTEQ	TRBV19	TRBJ2-7	1.11	1.25
NYGGSQGNL	TRAV25	TRAJ42	SLRSTGEL	TRBV19	TRBJ2-2	1.11	1.25
MTNAGGTSYGKL	TRAV38-1	TRAJ52	SFGSHGY	TRBV19	TRBJ1-2	1.11	1.25
ASHENYGGSQGNL	TRAV27	TRAJ42	SLYSQDEQ	TRBV19	TRBJ2-1	1.11	1.25
PGSQGNL	TRAV27	TRAJ42	SIRSSYEQ	TRBV19	TRBJ2-7	2.22	1.25
						17.78	52.50

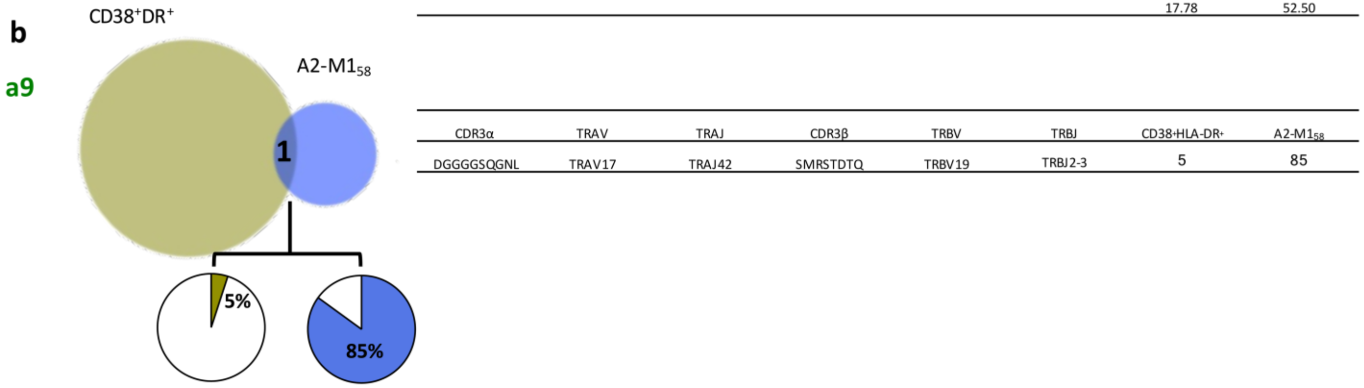

CDR3α	TRAV	TRAJ	CDR3β	TRBV	TRBJ	CD38+HLA-DR+	A2-M1$_{58}$
DGGGGSQGNL	TRAV17	TRAJ42	SMRSTDTQ	TRBV19	TRBJ2-3	5	85

Supplementary Figure 3. **TCRαβ repertoire sharing between M1$_{58}$$^{+}$CD8^{+} T cells and CD38^{+}HLA-DR^{+}CD8^{+} T cells in patients a79 and a9.** As shown for HLA-A*02:01-expressing patients **(a)** a79 and **(b)** a9, common TCRαβ clonotypes were detected in both A2-M1$_{58}$$^{+}$CD8^{+} T cells and CD38^{+}HLA-DR^{+}CD8^{+} TCRαβ repertoires. Proportion of TCRαβ sharing between A2-M1$_{58}$$^{+}$CD8^{+} T cells and CD38^{+}HLA-DR^{+}CD8^{+} T cells takes into consideration the frequency of each of the common TCRαβ clonotypes. Venn diagrams depicting shared TCRαβ clonotypes between CD38^{+}HLA-DR^{+} TCRαβ and A2-M1$_{58}$ datasets are shown. Pie chart represents % contribution of the shared TCRαβ clonotypes within CD38^{+}HLA-DR^{+} TCRαβ (olive green) and A2-M1$_{58}$ TCRαβ (blue) datasets. Shared TCRαβ clonotypes are listed in the corresponding tables.

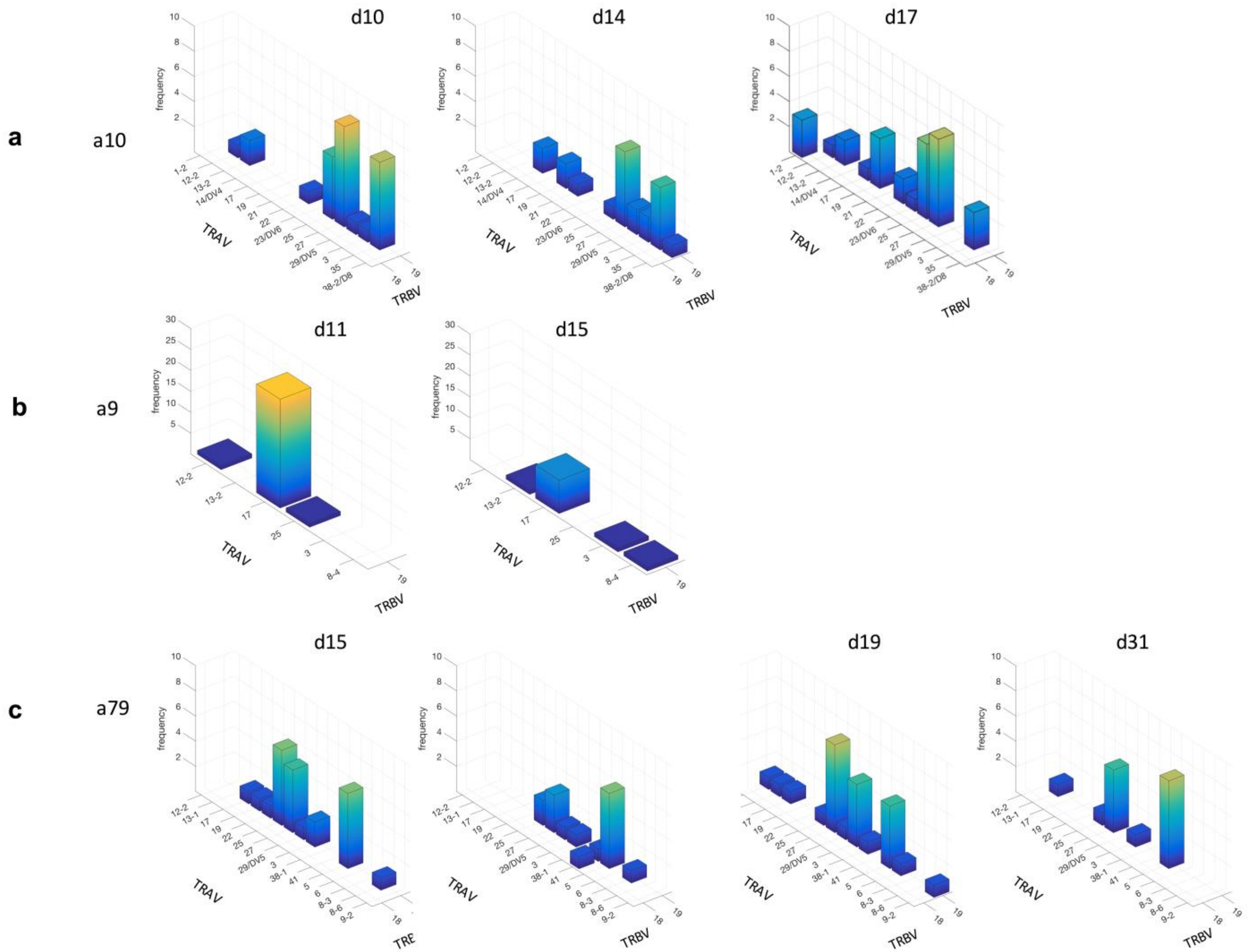

Supplementary Figure 4. **Restricted usage of TCRα-TCRβ pairings within A2-M1$_{58}^{+}$CD8^{+} T cells in H7N9-infected patients.** The frequency of TCRαβ clonotypes within TRAV-TRBV paired segments is shown for HLA-A*02:01-expressing patients: **(a)** 10, **(b)** a9, **(c)** a79. Longitudinal data across different time-points is shown.

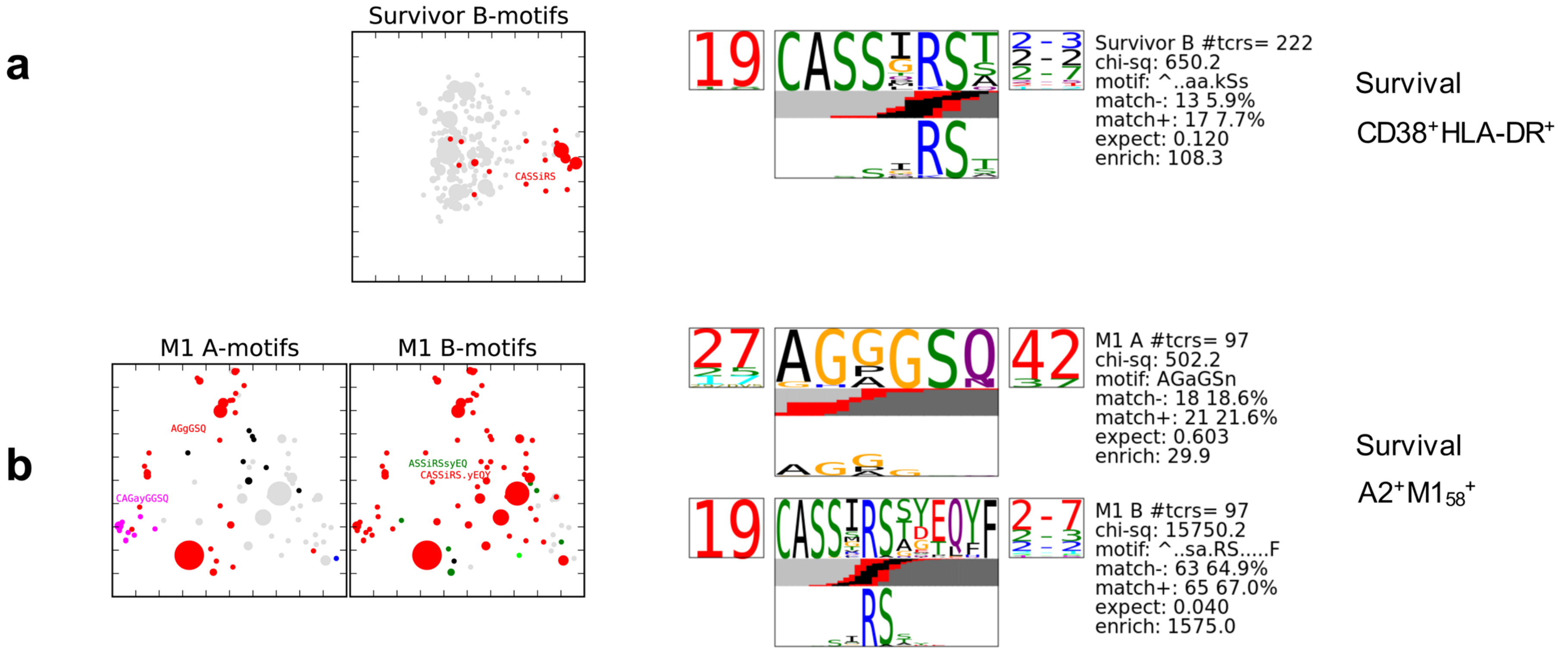

Supplementary Figure 5. **Motif enrichment analysis for survival A2-M1$_{58}^{+}$CD8^{+} and CD38^{+}HLA-DR^{+}CD8^{+} T cells.** Top enriched motifs were identified in the survival group for **(a)** CD38^{+}HLA-DR^{+}CD8^{+} and **(b)** A2-M1$_{58}^{+}$CD8^{+} TCRαβ clonotypes as the TRBV19 CDR3β was predominantly associated with the A2-M1$_{58}$ epitope. The top-scoring CDR3α and CDR3β sequence motifs are shown. No motifs were identified amongst the fatal CD38^{+}HLA-DR^{+}CD8^{+} TCRαβ clonotypes.

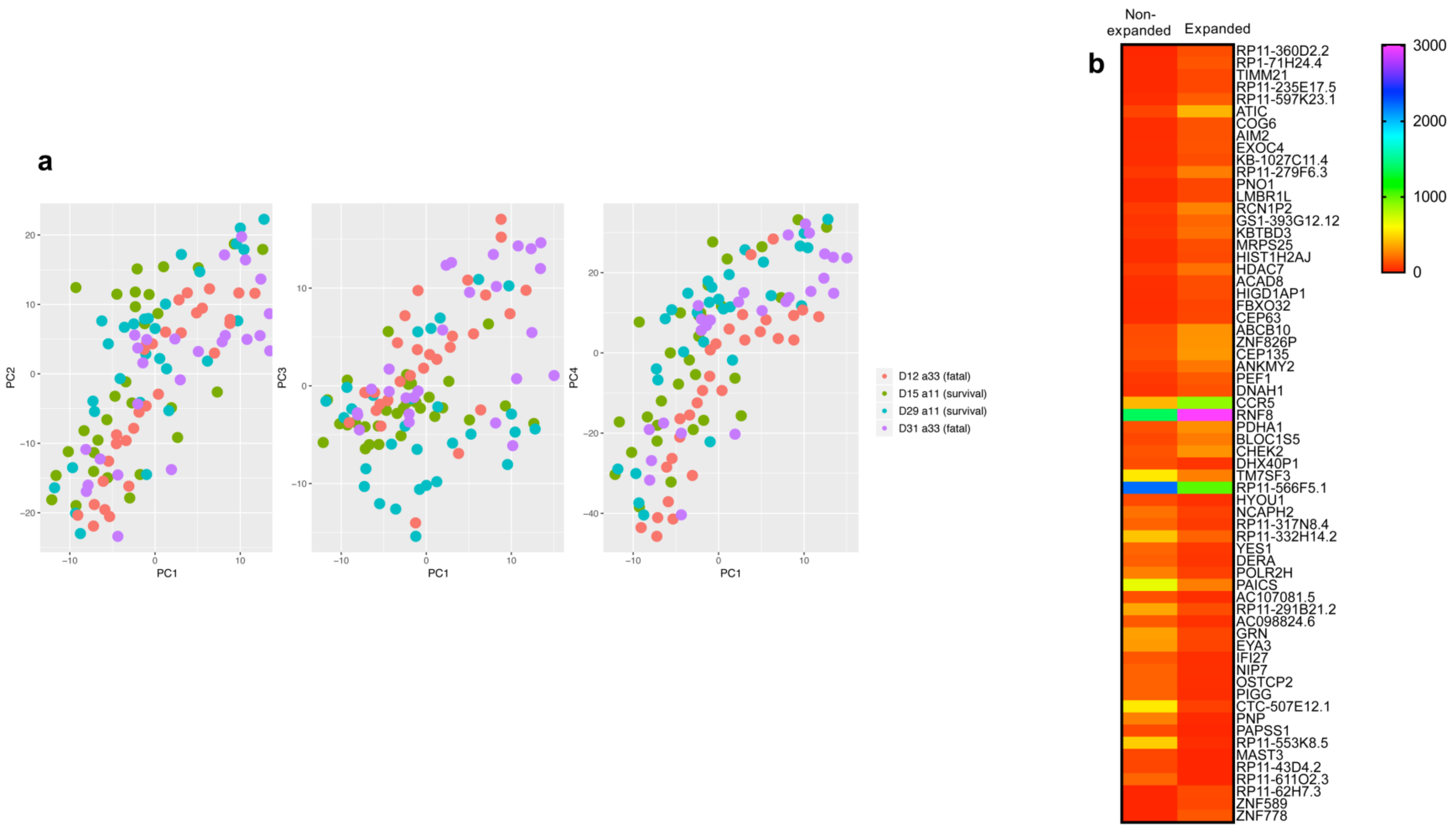

Supplementary Figure 6. **Single-cell RNAseq profiles for CD38$^+$HLA-DR$^+$CD8$^+$ T cells.** Single-cell RNAseq (scRNAseq) was performed on CD38$^+$HLA-DR$^+$CD8$^+$ T cells from longitudinal samples from two subjects, surviving a11 (d15, d29) and fatal a33 (d12, d31) patients. **(a)** tSNE analysis (PCA) of CD38$^+$HLA-DR$^+$CD8$^+$ T cells across the two patients is shown. **(b)** Heatmap of differentially expressed genes between all expanded and non-expanded TCRαβ clonotypes as shown in Fig.6.

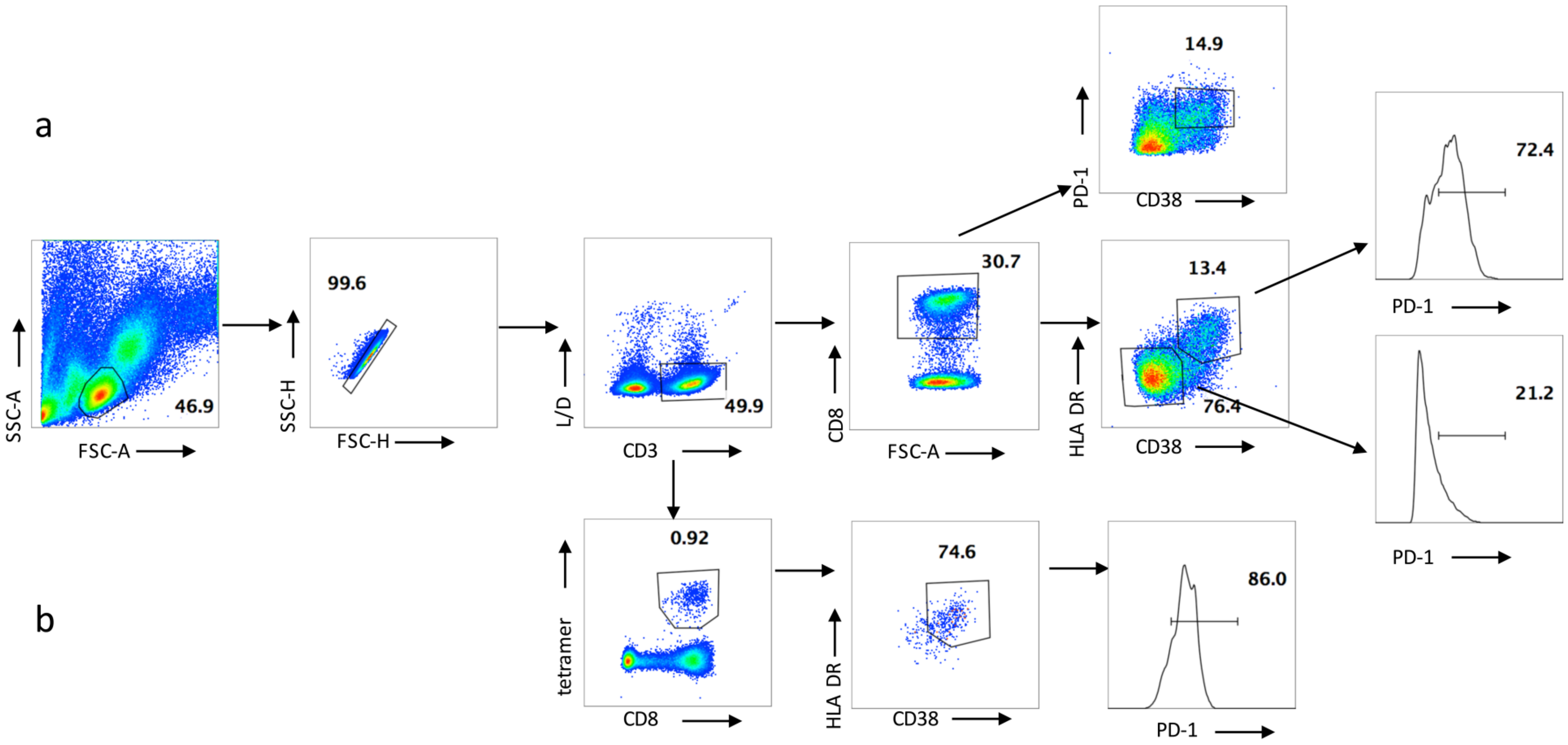

Supplementary Figure 7. Gating strategy for (a) CD38^{+}HLA-DR^{+}CD8^{+} T cells and (b) tetramer-positive A2/M1$_{58}$$^{+}$CD8^{+} T cells. PBMCs were stained with Live-dead Aqua 525 and a panel of antibodies: anti-CD3-PB, anti-CD8-FITC, anti-CD27-PE-Cy7, anti-CD45RA-APC-Cy7, anti-HLA-DR-ECD, anti-CD38-PerCPCy5.5, anti-PD-1-APC. Lymphocytes were washed, acquired on an LSR Fortessa with FACS Diva software (Becton Dickinson) and analyzed with FlowJo software. Gating strategy in (a) was used in Figs. 1, 2, 4, 5, 6; while gating strategy in (b) was used for Figs 3, 5.

Supplementary Table 1. Frequencies of CDR3αβ clonotypes within $A2^{+}M1_{58}^{+}CD8^{+}$ T cells in patient a9 at d11 and d15 after hospital admission.

Clone identifier	CDR3α	TRAV	TRAJ	CDR3β	TRBV	TRBJ	D11	D15
**A**	**DGGGGSQGNL**	**TRAV17**	**TRAJ42**	**SMRSTDTQ**	**TRBV19**	**TRBJ2-3**	**89%**	**73%**
B	QGSQGNL	TRAV12-2	TRAJ42	SFRSSYEQ	TRBV19	TRBJ2-7	4%	
C	DGGGGSQGNL	TRAV17	TRAJ42	SRRSTDTQ	TRBV19	TRBJ2-3	4%	
D	NYGGSQGNL	TRAV25	TRAJ42	SMRSTGEL	TRBV19	TRBJ2-2	4%	
E	LMDSNYQL	TRAV13-2	TRAJ33	GVRSGEVEQ	TRBV19	TRBJ2-1		9%
F	GGDGGSQGNL	TRAV3	TRAJ42	SIRSSYEQ	TRBV19	TRBJ2-7		9%
G	SDGENARL	TRAV8-4	TRAJ31	SIRSSYEQ	TRBV19	TRBJ2-7		9%
**# of CDR3αβ pairs**							**28**	**11**

*αβ CDR3 clonotypes found across all the time-points (depicted also in blue). **RS** motif is shown in pink.

Supplementary Table 2. Frequencies of CDR3αβ clonotypes within A2[+]M1$_{58}$[+]CD8[+] T cells in patient a10 at d10, d14 and d17 after hospital admission.

Clone identifier	CDR3α	TRAV	TRAJ	CDR3β	TRBV	TRBJ	D10	D14	D17
A	PSEPGDSNYQL	TRAV35	TRAJ33	SGLSNQPQ	TRBV19	TRBJ1-5	20%	14%	7%
B	ADGSGNTGKL	TRAV27	TRAJ37	SIRSAYEQ	TRBV19	TRBJ2-7	8%	10%	10%
C	QRGDGGSQGNL	TRAV35	TRAJ42	SIVSNQPQ	TRBV19	TRBJ1-5	8%		
D	IPVRNGRMNYGGSQGNL	TRAV29/DV5	TRAJ42	SSRSGIEQ	TRBV19	TRBJ2-7	4%	10%	
E	TPSNTGKL	TRAV22	TRAJ37	SIRSAYEQ	TRBV19	TRBJ2-7	4%		7%
F	NYGGSQGNL	TRAV25	TRAJ42	SIRSTGEL	TRBV19	TRBJ2-2	4%		7%
G	NSGGGSQGNL	TRAV13-2	TRAJ42	SFRATDTQ	TRBV19	TRBJ2-3	4%		3%
H	PGSNTGKL	TRAV27	TRAJ37	SIRSSYEQ	TRBV19	TRBJ2-7	4%		3%
I	IRSTL	TRAV13-2	TRAJ11	SIRSAYEQ	TRBV19	TRBJ2-7	4%		
J	ADGSSNTGKL	TRAV27	TRAJ37	SIRSAYEQ	TRBV19	TRBJ2-7	4%		
K	AGSQGNL	TRAV27	TRAJ42	SIRSSYEQ	TRBV19	TRBJ2-7	4%		
L	ASGIHNTDKL	TRAV27	TRAJ34	SMRSAYEQ	TRBV19	TRBJ2-7	4%		
M	GGSSNTGKL	TRAV27	TRAJ37	SSRSGWEQ	TRBV19	TRBJ2-7	4%		
N	PGSNTGKF	TRAV27	TRAJ37	SIRSSYEQ	TRBV19	TRBJ2-7	4%		
O	TYGGSQGNL	TRAV25	TRAJ42	SIRSTGEL	TRBV19	TRBJ2-2	4%		
P	AHGGSQGNL	TRAV25	TRAJ42	SGRSTDTQ	TRBV19	TRBJ2-3	4%		
Q	RDLKANAGKS	TRAV3	TRAJ27	SDISITEA	TRBV19	TRBJ1-1	4%		
R	WGSQGNL	TRAV25	TRAJ42	SSRSGNEQ	TRBV19	TRBJ2-1	4%		
S	QGSQGNL	TRAV12-2	TRAJ42	SSRSSYEQ	TRBV19	TRBJ2-7	4%		
T	SEVQLMDSNYQL	TRAV19	TRAJ33	SIYSGQPQ	TRBV19	TRBJ1-5		5%	10%
U	GGSQGNL	TRAV27	TRAJ42	SIRSSYEQ	TRBV19	TRBJ2-7		5%	3%
V	AVGSSNTGKL	TRAV27	TRAJ37	SIRSAYEQ	TRBV19	TRBJ2-7		5%	
W	DDQGGKL	TRAV27	TRAJ23	SIRSSYEQ	TRBV19	TRBJ2-7		5%	
X	QADRGSTLGRL	TRAV27	TRAJ18	SMYSINIQ	TRBV19	TRBJ2-4		5%	
Y	RGGNAGKS	TRAV35	TRAJ27	SGYSINEQ	TRBV19	TRBJ2-1		5%	
Z	SEDGNDYKL	TRAV19	TRAJ20	GFPYEQ	TRBV19	TRBJ2-7		5%	
AA	RDFEDSGNTPL	TRAV14/DV4	TRAJ29	STYSRDTQ	TRBV19	TRBJ2-3		5%	
AB	REGMEYGNKL	TRAV14/DV4	TRAJ47	SIFSTPNQPQ	TRBV19	TRBJ1-5		5%	
AC	NPGNTGKL	TRAV21	TRAJ37	SSYGSYEQ	TRBV19	TRBJ2-7		5%	
AD	RDGTGANNL	TRAV3	TRAJ36	GGISTDTQ	TRBV19	TRBJ2-3		5%	
AE	RDLETNAGKS	TRAV3	TRAJ27	STTSGTEQ	TRBV19	TRBJ2-1		5%	
AF	SYGGSQGNL	TRAV25	TRAJ42	SIRSTDTQ	TRBV19	TRBJ2-3		5%	
AG	SLSAGGTSYGKL	TRAV38-2/D8	TRAJ52	SIGVYGY	TRBV19	TRBJ1-2		5%	
AH	VEAEYGNKL	TRAV1-2	TRAJ47	SLTGTVYEQ	TRBV18	TRBJ2-7			10%
AI	AGNTGKL	TRAV27	TRAJ37	SVRSSYEQ	TRBV19	TRBJ2-7			7%
AJ	NPSNTGKL	TRAV23/DV6	TRAJ37	SIRAAYEQ	TRBV19	TRBJ2-7			3%
AK	NNGGGSQGNL	TRAV13-2	TRAJ42	SGRAGGEL	TRBV19	TRBJ2-2			3%

**AL**	GGSQGNL	TRAV12-2	TRAJ42	SIRSSYEQ	TRBV19	TRBJ2-7			3%
**AM**	NYGGSQGNL	TRAV25	TRAJ42	SRRSTDTQ	TRBV19	TRBJ2-3			3%
**AN**	NYGGSQGNL	TRAV35	TRAJ42	SMRGSDTQ	TRBV19	TRBJ2-3			3%
**AO**	PGSNTGKL	TRAV25	TRAJ37	SIRSSQPQ	TRBV19	TRBJ1-5			3%
**AP**	SYGGSQGNL	TRAV25	TRAJ42	SIRSHDEQ	TRBV19	TRBJ2-1			3%
**AQ**	SYGGSQGNL	TRAV25	TRAJ42	SIRSTDTQ	TRBV19	TRBJ2-3			3%
**AR**	VEGSYIP	TRAV19	TRAJ6	GPHGGGNNEQ	TRBV19	TRBJ2-1			3%
**AS**	DAMPPGGNTGKL	TRAV17	TRAJ37	SFYSNQPQ	TRBV19	TRBJ1-5			3%
**of CDR3$\alpha\beta$ pairs**							**25**	**21**	**30**

$\alpha\beta$ CDR3 clonotypes found across all the time-points (depicted also in blue); #$\alpha\beta$ CDR3 clonotypes found at >1 time-point (depicted in red). RS motif is shown in pink. and $\alpha\beta$ CDR3 clonotypes found at >1 in one time-point (depicted in green).

Underlining is the difference in residues to the main referenced public motifs in that colour block

Supplementary Table 3. Frequencies of CDR3αβ clonotypes within $A2^{+}M1_{58}^{+}CD8^{+}$ T cells in patient a79 at d15, d17, d19 and d31 after hospital admission.

Clone identifier	CDR3α	TRAV	TRAJ	CDR3β	TRBV	TRBJ	D15	D17	D19	D31
A	DNQGGKL	TRAV5	TRAJ23	SGRSADTQ	TRBV19	TRBJ2-3	25%	38%	16%	40%
B	NYGGSQGNL	TRAV25	TRAJ42	SIRSTGEL	TRBV19	TRBJ2-2	8%	6%		
C	SGIGGSQGNL	TRAV3	TRAJ42	STRSSYEQ	TRBV19	TRBJ2-7	4%	6%	8%	
D	AFGSSNTGKL	TRAV27	TRAJ37	SIRSAYEQ	TRBV19	TRBJ2-7	4%	6%	4%	7%
E	SSSNTGKL	TRAV3	TRAJ37	SGRSQETQ	TRBV19	TRBJ2-5	4%		8%	7%
F	AGGGSQGNL	TRAV27	TRAJ42	SGRSTDTQ	TRBV19	TRBJ2-3	4%			7%
G	AGSQGNL	TRAV27	TRAJ42	SSRSSYEQ	TRBV19	TRBJ2-7	4%			7%
H	LYGGSQGNL	TRAV25	TRAJ42	SILSQQPQ	TRBV19	TRBJ1-5	4%			
I	SALRGMDSNYQL	TRAV29/DV5	TRAJ33	SIFAHSSQPQ	TRBV19	TRBJ1-5	4%			
J	SSNTGKL	TRAV8-6	TRAJ37	SIRSSYEQ	TRBV19	TRBJ2-7	4%			
K	ADSSSNTGKL	TRAV27	TRAJ37	SIRSAYEQ	TRBV19	TRBJ2-7	4%			
L	AGGGSQGNL	TRAV25	TRAJ42	SKRSSDTQ	TRBV19	TRBJ2-3	4%			
M	AGGGSQGNL	TRAV27	TRAJ42	SMRSAGEL	TRBV19	TRBJ2-2	4%			
N	NYGGSQGNL	TRAV25	TRAJ42	SQRSTDTQ	TRBV19	TRBJ2-3	4%			
O	TYGGSQGNL	TRAV25	TRAJ42	SIRSTGEL	TRBV19	TRBJ2-2	4%			
P	SEASAGGTSYGKL	TRAV19	TRAJ52	SMAGGPGNEQ	TRBV19	TRBJ2-1	4%			
Q	DGSSSNTGKL	TRAV17	TRAJ37	SIYSGGYNEQ	TRBV19	TRBJ2-1	4%			
R	DVSDRGSTLGRL	TRAV22	TRAJ18	SGTSFTEQ	TRBV19	TRBJ2-7	4%			
S	GGSQGNL	TRAV27	TRAJ42	SIRSSYEQ	TRBV19	TRBJ2-7		13%		
T	SAGGGSQGNL	TRAV29/DV5	TRAJ42	SIRASYEQ	TRBV19	TRBJ2-7		6%	4%	
U	NYGGSQGNL	TRAV25	TRAJ42	SLRSTGEL	TRBV19	TRBJ2-2		6%		
V	LLQGAQKL	TRAV41	TRAJ54	SFGMHGY	TRBV19	TRBJ1-2		6%		
W	GGSQGNL	TRAV8-3	TRAJ42	SMISINEQ	TRBV19	TRBJ2-1		6%		
X	SPMEYGNKL	TRAV41	TRAJ47	SPVGIGEA	TRBV18	TRBJ1-1		6%		
Y	DGGGGSQGNL	TRAV27	TRAJ42	SIRSTGEL	TRBV19	TRBJ2-2			8%	
Z	QGSQGNL	TRAV13-1	TRAJ42	STRSSETQ	TRBV19	TRBJ2-5			4%	7%
AA	MTNAGGTSYGKL	TRAV38-1	TRAJ52	SFGSHGY	TRBV19	TRBJ1-2			4%	
AB	AGSQGNL	TRAV27	TRAJ42	SIRSSYEQ	TRBV19	TRBJ2-7			4%	
AC	ASGSSNTGKL	TRAV27	TRAJ37	SIRSAYEQ	TRBV19	TRBJ2-7			4%	
AD	ASHENYGGSQGNL	TRAV27	TRAJ42	SLYSQDEQ	TRBV19	TRBJ2-1			4%	
AE	MEYGNKL	TRAV3	TRAJ47	SIRSTDTQ	TRBV19	TRBJ2-3			4%	
AF	PGSQGNL	TRAV27	TRAJ42	SIRSSYEQ	TRBV19	TRBJ2-7			4%	
AG	DMGGGSQGNL	TRAV6	TRAJ42	SIRAADTQ	TRBV19	TRBJ2-3			4%	
AH	QGSQGNL	TRAV9-2	TRAJ42	SSRSGVEQ	TRBV19	TRBJ2-7			4%	
AI	NYGGSQGNL	TRAV25	TRAJ42	SMRSTGEL	TRBV19	TRBJ2-2			4%	
AJ	ETGNQF	TRAV5	TRAJ49	SGRSAGEL	TRBV19	TRBJ2-2			4%	

AK	DAGGGSQGNL	TRAV17	TRAJ42	MGLAGLNEQ	TRBV19	TRBJ2-1			**4%**	
AL	NRGGGSQGNL	TRAV12-2	TRAJ42	SIRSSYEQ	TRBV19	TRBJ2-7			**4%**	
AM	SIPAGANSKL	TRAV5	TRAJ56	SRYHTDTQ	TRBV19	TRBJ2-3				**7%**
AN	AGGGSQGNL	TRAV27	TRAJ42	SIRSTDTQ	TRBV19	TRBJ2-3				**7%**
AO	AGGGSQGNL	TRAV27	TRAJ42	STRSTDTQ	TRBV19	TRBJ2-3				**7%**
AP	SYGGSQGNL	TRAV25	TRAJ42	STRSTSPG	TRBV19	TRBJ1-5				**7%**
**# of CDR3ab pairs**							**24**	**16**	**25**	**15**

*αβ CDR3 clonotypes found across all the time-points (depicted also in blue); #*αβ CDR3 clonotypes found at >1 time-point (depicted in red). **RS** motif is shown in pink. and αβ CDR3 clonotypes found at >1 in one time-point (depicted in green).

Supplementary Table 4. Summary of all the samples used in TCR analysis

Group	Individual	Sample	Timepoint	Epitope	HLA-A	HLA-A	HLA-B	HLA-B	Discharged after days of onset or death
survival	A10	A10 D10	Day10	$M1_{58}^{+}$	0201	3201	702	4001	18
		A10 D14	Day14						
		A10 D17	Day17						
	A9	a9 D11	Day11		0201	0207	1511	4002	23
		a9 D15	Day15						
	A79	a79 D15	Day15		0201	1101	1501	0603	31
		a79 D17	Day 17						
		a79 D19	Day19						
		a79 D31	Day31						
	A9	a9 D11	Day11	$DR^{+}CD38^{+}$	0201	0207	1511	4002	23
		a9 D15	Day15						
	A20	A20 D5	Day5		1102	2601	1518	1518	23
		A20 D17	Day17						
	A78	A78 D11	Day11		0207	2402	4601	4801	22
		A78 D17	Day17						
	A79	a79 D15	Day15		0201	1101	1501	5603	31
		a79 D17	Day 17						
		a79 D31	Day31						
	A11	A11 d9	Day9		3001	3101	1302	4006	33
		A11 D13	Day13						
		A11 D18	Day18						
fatal	A131	a131 D22	Day22	$DR^{+}CD38^{+}$	0301	1101	1302	3802	death
		a131 D27	Day27						
		a131 D32	Day32						

	a33 D8	Day8	0206	3101	4006	5601	death
A33	a33 D19	Day19					
	a33 D33	Day33					

Supplementary Table 5. Frequencies of CDR3αβ clonotypes within HLA-DR+CD38+ CD8+ T cells in patients a78, a20, a11, a9, a79, a131 and a33 at different time points after hospital admission.

**Donor a78 Clone identifier**	**CDR3α**	**TRAV**	**TRAJ**	**CDR3β**	**TRBV**	**TRBJ**	**D11**	**D17**
**A**	**NAQAAGNKL**	**TRAV8-1**	**TRAJ17**	**SPHWTNTDTQ**	**TRBV11-2**	**TRBJ2-3**	**10%**	
**B**	**PWSSGTYKY**	**TRAV35**	**TRAJ40**	**SSPSGTSGSYEQ**	**TRBV7-8**	**TRBJ2-7**	**5%**	**4%**
**C**	**RRRGLGGGGFKT**	**TRAV21**	**TRAJ9**	**SKTGGQAGEL**	**TRBV9**	**TRBJ2-2**	**5%**	**4%**
D	KESGSARQL	TRAV13-1	TRAJ22	SVRGGGSPSDTQ	TRBV7-9	TRBJ2-3	5%	
E	LIQGAQKL	TRAV29/DV5	TRAJ54	SITTPLTGEL	TRBV30	TRBJ2-2	5%	
F	SYFGNEKL	TRAV13-1	TRAJ48	SGARGGNEQ	TRBV7-9	TRBJ2-1	5%	
G	DTGNQF	TRAV39	TRAJ49	TVFFGTGGAYGY	TRBV6-1	TRBJ1-2	5%	
H	GAGGFKT	TRAV12-2	TRAJ9	SDLSTDTQ	TRBV10-1	TRBJ2-3	5%	
I	SDLGNTGGFKT	TRAV19	TRAJ9	SYSKGGPNTGEL	TRBV6-5	TRBJ2-2	5%	
J	DNTDKL	TRAV6	TRAJ34	KGTGGYNEQ	TRBV14	TRBJ2-1	5%	
K	ATDSWGKF	TRAV8-6	TRAJ24	SWDRDDSPL	TRBV25-1	TRBJ1-6	5%	
L	GAFAGGGKL	TRAV8-3	TRAJ23	SQGQYEGTVYGY	TRBV3-1	TRBJ1-2	5%	
M	IGASGTYKY	TRAV12-1	TRAJ40	SLLAGGEETQ	TRBV28	TRBJ2-5	5%	
N	RDAGNNHIQ	TRAV3	TRAJ38	SVWGGEGADTQ	TRBV9	TRBJ2-3	5%	
O	RGRSGGYQKV	TRAV19	TRAJ13	SLRGRAEETQ	TRBV27	TRBJ2-5	5%	
P	RVAQSAAGNKL	TRAV1-2	TRAJ17	SQESGTSGSTDTQ	TRBV4-1	TRBJ2-3	5%	
Q	TEGGSEKL	TRAV21	TRAJ57	GRSLEL	TRBV7-9	TRBJ1-4	5%	
R	DVRGGYNKL	TRAV4	TRAJ4	SLKGTGKADEQ	TRBV5-4	TRBJ2-1	5%	
S	VPGARL	TRAV17	TRAJ31	SVTGNTEA	TRBV9	TRBJ1-1	5%	
T	SDQRYQL	TRAV8-2	TRAJ33	SLYAENTGEL	TRBV11-2	TRBJ2-2	5%	
**U**	**ETSGSRL**	**TRAV39**	**TRAJ58**	**AAPGARVRYEQ**	**TRBV29-1**	**TRBJ2-7**		**11%**
V	NNTGFQKL	TRAV13-2	TRAJ8	SLEASGGVTDTQ	TRBV5-6	TRBJ2-3		4%
W	LEGSTLGRL	TRAV1-2	TRAJ18	SVDSTTYNEQ	TRBV9	TRBJ2-1		4%
X	SEEAAGNKL	TRAV19	TRAJ17	SIFITNGQPNTEA	TRBV19	TRBJ1-1		4%
Y	SGVSGGGADGL	TRAV14/DV4	TRAJ45	SLGVDTTNEKL	TRBV28	TRBJ1-4		4%
Z	VRRGGSNYKL	TRAV41	TRAJ53	SRGAGFYNEQ	TRBV7-2	TRBJ2-1		4%
AA	NQGGKL	TRAV21	TRAJ23	RTPTGANEKL	TRBV6-3	TRBJ1-4		4%
AB	EAIIQGAQKL	TRAV36/DV7	TRAJ54	SLGQGYRSPL	TRBV5-8	TRBJ1-6		4%
AC	EGGGTSYGKL	TRAV20	TRAJ52	SQVTSSDTQ	TRBV4-1	TRBJ2-3		4%
AD	KRGRAGSYQL	TRAV21	TRAJ28	SVRLAGGPRQAYEQ	TRBV30	TRBJ2-7		4%
AE	NNMMGRGSTLGRL	TRAV12-2	TRAJ18	SQVGSNQPQ	TRBV3-1	TRBJ1-5		4%
AF	NSGGSNYKL	TRAV12-2	TRAJ53	SNFRGLGSGANVL	TRBV18	TRBJ2-6		4%
AG	PRMEYGNKL	TRAV21	TRAJ47	SPRDLPGEQ	TRBV18	TRBJ2-7		4%
AH	RDIGGDM	TRAV3	TRAJ43	SFGGADTQ	TRBV7-3	TRBJ2-3		4%

AI	RDPPKAAGNKL	TRAV1-1	TRAJ17	SLGAGDEKL	TRBV19	TRBJ1-4		4%
AJ	RMNRDDKI	TRAV1-2	TRAJ30	SQRADNTDTQ	TRBV4-2	TRBJ2-3		4%
AK	RSSYNTDKL	TRAV1-2	TRAJ34	SHRADNTDTQ	TRBV4-2	TRBJ2-3		4%
AL	SDRSLRDDKI	TRAV3	TRAJ30	SLGQGGTPL	TRBV7-6	TRBJ1-6		4%
AM	SEANQAGTAL	TRAV8-6	TRAJ15	NRRQGST	TRBV2	TRBJ1-1		4%
AN	SVLSQDTDKL	TRAV8-6	TRAJ34	SYQGGGAGY	TRBV27	TRBJ1-2		4%
AO	VRMDSNYQL	TRAV8-3	TRAJ33	TGQGLSYEQ	TRBV5-5	TRBJ2-7		4%
AP	RSTPGTASKL	TRAV38-2/DV8	TRAJ44	SDSPQDRLNTGEL	TRBV9	TRBJ2-2		4%
AQ	EGGDYKL	TRAV30	TRAJ20	SQRYQGRAGNTI	TRBV4-3	TRBJ1-3		4%
**# of CDR3αβ pairs**							**21**	**27**

Supplementary Table 6. Details of the donors used in the study.

Donor code	Age	HLA-A	HLA-B	Figure	Discharged at days after disease onset or death
**Healthy donor**					
BP90	NA	02:01, 24[+]	07, 40+	Fig.3b	NA
BP39	56	02:01, 02:01	07:02,44:01	Fig.3b	NA
BP9	22	02:01,68:01	44:02, 51:01	Fig.3b	NA
BP16	25	02:01, 68:01	35:01, 3901	Fig.3b	NA
BP17	25	02:01,1101	3501, 3901	Fig.3b	NA
**H7N9 survival patients**					
a11	75	30:01, 11:01	13:02, 40:06	Fig.1b-i, Fig2b, 2d,2f, Fig4a, Fig5a-d, Fig6a-d	33
a20	81	11:02, 26:01	15:18, 15:18	Fig.1b-i, Fig2b-f, Fig4a, Fig5a-d, Fig6a-d	23
a78	74	02:07, 24:02	46:01,48:01	Fig.1b-i, Fig2b-f, Fig4a, Fig5a-d, Fig6a-d	22
a10	65	02:01, 32:01	07:02, 40:01	Fig.1b-i, Fig2b-f, Fig3a,3c,	18
a9	67	02:01, 02:07,	15:11, 40:02	Fig.1b-i, Fig2b-f, Fig3a,3c, Fig4b, Fig5a-d, Fig6a-d	23
a79	78	02:01, 11:01	15:01, 56:03	Fig.1a-i, Fig2b-f, Fig3a,3c, Fig4b, Fig5a-d, Fig6a-d	31
**H7N9 fatal patients**					
a131	79	03:01, 11:01	13:02, 38:02	Fig.1b-i, Fig2b-f, Fig4c, Fig5a-d, Fig6a-d	62
a33	56	02:06, 31:01	40:06, 56:01	Fig.1a-i, Fig2b-f, Fig4c, Fig5a-d, Fig6a-d	76

Molecular resolution of HLA class I antigens (4-digit) are shown where available. NA: Not applicable;

Supplementary Table 7. Frequencies of expanded (blue) and non-expanded (black) CDR3αβ clonotypes within CD38^{+}HLA-DR^{+} CD8^{+} T cells in patients a11and a33 at different time-points after hospital admission, as detected by single-cell RNAseq

**a11 patient**						D15 ScRNA seq		D29 ScRNA seq	
**TRAV**	**TRAJ**	**CDR3α**	**TRBV**	**TRBJ**	**CDR3β**	#	%	#	%
TRAV14/DV4	TRAJ31	TVGNARL	TRBV7-3	TRBJ2-5	SPDPGETQ	2	9%		
TRAV14/DV4	TRAJ52	REGLDGTSYGKL	TRBV5-6	TRBJ1-2	TPAGSLYGY	2	9%	4	22%
TRAV16	TRAJ18	MDRRGSTLGRL	TRBV11-2	TRBJ2-2	SPLGTHPNTGEL			3	16%
TRAV17	TRAJ42	PTGRDGGSQGNL	TRBV11-2	TRBJ2-2	SPLGTHPNTGEL			3	16%
TRAV29/DV5	TRAJ39	SGSAGNML	TRBV5-1	TRBJ1-3	SLDFRNTI			2	11%
TRAV38-2/DV8	TRAJ4	FSGGYNKL	TRBV4-1	TRBJ1-1	SQDRYRMNTEA			2	11%
TRAV17	TRAJ22	GDSGSARQL	TRBV4-1	TRBJ2-3	SHQPLAGDTQ	2	9%		
TRAV17	TRAJ45	DGEYSGGGADGL	TRBV7-9	TRBJ1-1	SLQGWTEA	1	4%		
TRAV17	TRAJ6	DDDYKLSFGAGTTVTVRARGSYIP	TRBV19	TRBJ1-1	SIGRGNTEA	1	4%		
TRAV17	TRAJ20	DDDYKL	TRBV19	TRBJ1-1	SIGRGNTEA	1	4%		
TRAV24	TRAJ45	IDSGGGADGL	TRBV19	TRBJ1-1	SIHRVNTEA	1	4%		
TRAV14/DV4	TRAJ52	REGLDGTSYGKL	TRBV5-6	TRBJ1-2	TPAGSLYGY	1	4%		
TRAV27	TRAJ26	ADNYGQNF	TRBV18	TRBJ1-2	SRYGSPLSGY	1	4%		
TRAV40	TRAJ33	NYQL	TRBV18	TRBJ1-2	SRYGSPLSGY	1	4%		
TRAV5	TRAJ9	ENTGGFKT	TRBV2	TRBJ1-2	THRGGGY	1	4%		
TRAV17	TRAJ9	DPDNTGGFKT	TRBV2	TRBJ1-4	RETGGNEKL	1	4%		
TRAV3	TRAJ18	RDVGGSTLGRL	TRBV7-9	TRBJ1-4	SLGVGEKL	1	4%		
TRAV12-2	TRAJ4	ISGGYNKL	TRBV11-2	TRBJ1-5	SWGQGGNQPQ	1	4%		
TRAV14/DV4	TRAJ11	RASGYSTL	TRBV6-5	TRBJ2-1	SKFGPGNNEQ	1	4%		
TRAV1-2	TRAJ23	RDMIYNQGGKL	TRBV9	TRBJ2-2	SVAEAGEL	1	4%		
TRAV8-6	TRAJ42	TIWGGSQGNL	TRBV2	TRBJ2-2	SVSGAGGEL	1	4%		
TRAV29/DV5	TRAJ4	KGVSGGYNKL	TRBV2	TRBJ2-6	SPRGFSAGANVL	1	4%		

TRAV14/DV4	TRAJ58	RETSGSRL	TRBV2	TRBJ2-7	SEFFGSGLSSYE Q	1	4%		
TRAV8-1	TRAJ11	NAGYSTL	TRBV27	TRBJ2-7	SGTGGLSHEQ	1	4%		
TRAV14/DV4	TRAJ52	REGLDGTSYGKL	TRBV5-6	TRBJ1-2	TPAGSLYGY			1	5%
TRAV12-2	TRAJ26	SGQNF	TRBV5-5	TRBJ2-1	SFDLYNEQ			1	5%
TRAV19	TRAJ57	IPQLGQGGSEKL	TRBV7-9	TRBJ1-1	SLVVWGRNTEA			1	5%
TRAV21	TRAJ43	ISDM	TRBV7-2	TRBJ1-2	SLSPSDGY			1	5%
TRAV19	TRAJ48	SEAFGDFGNEKL	TRBV7-9	TRBJ2-1	SIWVGTGSQGEQ			1	5%
TRAV38-2/DV8	TRAJ13	YSGGYQKV	TRBV4-1	TRBJ2-7	SQDLYRASYEQ			1	5%
						23		19	

a33 patient						D12 ScRNA seq		D31 ScRNA seq	
TRAV	TRAJ	CDR3α	TRBV	TRBJ	CDR3β	#	f%	#	f%
TRAV3	TRAJ32	RDFRYGGATNKL	TRBV5-6	TRBJ2-7	SGAGTDFYEQ	1	3%	1	4%
TRAV13-2	TRAJ30	KDDRDDKI	TRBV6-6	TRBJ2-3	SYPEGLAGSTWGDT Q	3	10%	1	4%
TRAV14/DV4	TRAJ53	REGSRMGGGSNYK L	TRBV19	TRBJ1-1	SIYENTEA	2	7%		
TRAV38-2/DV8	TRAJ47	EEYGNKL	TRBV24-1	TRBJ2-3	RGVSRGDTDTQ	3	10%	1	4%
TRAV38-2/DV8	TRAJ40	GTSGTYKY	TRBV4-1	TRBJ2-1	SQDLYRGLGEQ	3	10%		
TRAV26-1	TRAJ30	RDNRDDKI	TRBV6-6	TRBJ2-7	SYSEKTGDEQ	1	3%		
TRAV14/DV4	TRAJ42	DGGSQGNL	TRBV4-1	TRBJ2-1	SQAPSGSYNEQ	1	3%		
TRAV21	TRAJ57	RLITQGGSEKL	TRBV14	TRBJ2-7	SLYPGYEQ	1	3%		
TRAV22	TRAJ42	PGVYGGSQGNL	TRBV7-9	TRBJ1-4	SLVADEKL	1	3%		
TRAV25	TRAJ53	RLYRVNRKL	TRBV19	TRBJ1-1	SIYENTEA	1	3%		
TRAV26-1	TRAJ42	GLNYGGSQGNL	TRBV7-7	TRBJ1-3	SFGQGAVGGNTI	1	3%		
TRAV26-2	TRAJ30	RDDRDDKI	TRBV6-6	TRBJ1-6	SYNEFSSYNSPL	1	3%		
TRAV27	TRAJ13	DHSGGYQKV	TRBV28	TRBJ2-7	SSPDPTRGHEQ	1	3%		
TRAV27	TRAJ40	ALASGTYKY	TRBV9	TRBJ2-3	SVSVTDTQ	1	3%		
TRAV27	TRAJ40	AAASGTYKY	TRBV9	TRBJ2-3	SISITDTQ	1	3%		
TRAV27	TRAJ40	AAASGTYKY	TRBV20-1	TRBJ2-7	RKFTDRTTYEQ	1	3%		
TRAV29/DV5	TRAJ49	SRGNQF	TRBV6-6	TRBJ1-6	SYNEFSSYNSPL	1	3%		
TRAV30	TRAJ43	NNNDM	TRBV5-6	TRBJ1-4	SYWTGPAATNEKL	1	3%		
TRAV38-2/DV8	TRAJ40	GTSGTYKY	TRBV4-1	TRBJ2-1	SQDLYRGLGEQ	1	3%		
TRAV8-1	TRAJ36	NPDRGANNL	TRBV7-2	TRBJ1-4	SLSSATNEKL	1	3%		
TRAV8-2	TRAJ47	SHPDYGNKL	TRBV19	TRBJ2-1	SIFAGLAAYNEQ	1	3%		
TRAV8-6	TRAJ30	SEPSDDKI	TRBV7-8	TRBJ2-7	SLERVAVYEQ	1	3%		
TRAV9-2	TRAJ30	EGRDDKI	TRBV5-4	TRBJ1-2	SLSGRVEIGY	1	3%		

TRAV14/DV4	TRAJ30	RVMEDRDDKI	TRBV25-1	TRBJ1-1	SDATAMGPNTEA		1	4%
TRAV1-2	TRAJ31	HDNNARL	TRBV7-2	TRBJ2-1	SPQDLEQ		1	4%
TRAV12-2	TRAJ28	TPGSYQL	TRBV7-8	TRBJ2-7	SLRTYEQ		1	4%
TRAV12-3	TRAJ42	SALGSQGNL	TRBV5-4	TRBJ1-1	SPGLYTEA		1	4%
TRAV12-3	TRAJ27	SDSANAGKS	TRBV29-1	TRBJ1-2	GGPRPGY		1	4%
TRAV12-3	TRAJ3	SAYSSASKI	TRBV7-2	TRBJ2-1	SPQDLEQ		1	4%
TRAV13-1	TRAJ4	SQHGGYNKL	TRBV20-1	TRBJ2-3	QRRGEITDTQ			
TRAV17	TRAJ29	DERNTPL	TRBV7-9	TRBJ2-7	SVGPGTGLYEQ		1	4%
TRAV17	TRAJ29	DERNTPL	TRBV6-4	TRBJ1-3	GRGKSEGTVNGNTI		1	4%
TRAV2	TRAJ24	EGGEKTTDSWGKF	TRBV7-8	TRBJ2-7	SLRTYEQ		1	4%
TRAV21	TRAJ49	LTNTGNQF	TRBV6-2	TRBJ1-2	SPGTGFYGY		1	4%
TRAV21	TRAJ30	RERDDKI	TRBV5-4	TRBJ2-7	SPDGDLLNEQ		1	4%
TRAV21	TRAJ47	AEYGNKL	TRBV6-5	TRBJ2-3	SSGQSTDTQ		1	4%
TRAV22	TRAJ45	TGGGADGL	TRBV11-2	TRBJ2-7	SLGTNYEQ		1	4%
TRAV22	TRAJ21	ELGFNKF	TRBV10-3	TRBJ2-5	TGGGETQ		1	4%
TRAV25	TRAJ25	YVGRKF	TRBV29-1	TRBJ2-1	TQGGTYNEQ			
TRAV25	TRAJ53	HSGGSNYKL	TRBV9	TRBJ2-7	SDGGTGVYEQ		1	4%
TRAV26-2	TRAJ45	RDPGAYSGGGADGL	TRBV29-1	TRBJ2-7	EDYTGQHYEQ		1	4%
TRAV35	TRAJ41	RENSGYAL	TRBV5-1	TRBJ1-1	KEGTEA		1	4%
TRAV35	TRAJ33	RGDSNYQL	TRBV10-2	TRBJ2-1	RRLTSGGNNEQ		1	4%
TRAV4	TRAJ20	GADYKL	TRBV4-3	TRBJ2-7	SHGLAGVSSYEQ		1	4%
TRAV5	TRAJ17	KQNKAAGNKL	TRBV7-9	TRBJ2-7	SPQRNYEQ		1	4%
TRAV8-3	TRAJ56	GGLAGANSKL	TRBV5-4	TRBJ1-1	SPGLYTEA		1	4%
TRAV8-3	TRAJ29	NSGNTPL	TRBV7-9	TRBJ2-5	SIQETQ		1	4%
TRAV8-4	TRAJ22	NSGSGSARQL	TRBV24-1	TRBJ1-5	SDWDRDQPQ		1	4%
TRAV8-6	TRAJ8	TQSRGFQKL	TRBV11-2	TRBJ2-3	SFGQGTDTQ		1	4%
						30	27	

Abbreviations: ND, not determined; X, different amino acid; #, unproductive due to out-of-frame shift.
αβ CDR3 clonotypes found at >1 during single-cell RNAseq analysis are marked in blue.

Supplementary Table 8. Primers used for the single-cell RT-PCR TCR amplification.

Primer Name	Seq	Primer Name	Seq
	External primers		Internal primers
**hTRACext (reverse)**	GACCAGCTTGACATCACAG	**hTRACint (reverse)**	TGTTGCTCTTGAAGTCCATAG
hTRBV2ext	TCGATGATCAATTCTCAGTTG	hTRBV2int	TTCACTCTGAAGATCCGGTC
hTRBV3ext	CAAAATACCTGGTCACACAG	hTRBV3int	AATCTTCACATCAATTCCCTG
hTRBV4ext	TCGCTTCTCACCTGAATG	hTRBV4int	CCTGCAGCCAGAAGACTC
hTRBV5-1ext	GATTCTCAGGKCKCCAGTTC	hTRBV5-1int	CTTGGAGCTGGRSGACTC
hTRBV5-5ext	GTACCAACAGGYCCTGGGT	hTRBV5-5int	TCTGAGCTGAATGTGAACG
hTRBV6-1ext	ACTCAGACCCCAAAATTCC	hTRBV6-1int	GTGTRCCCAGGATATGAACC
hTRBV6-4ext	ACTGGCAAAGGAGAAGTCC	hTRBV6-4int	TGGTTATAGTGTCTCCAGAGC
hTRBV7-1ext	TRTGATCCAATTTCAGGTCA	hTRBV7-1int	TCYACTCTGAMGWTCCAGCG
hTRBV7-4ext	CGSWTCTYTGCAGARAGGC	hTRBV7-4int	TGRMGATYCAGCGCACA
hTRBV9ext	GATCACAGCAACTGGACAG	hTRBV9int	GTACCAACAGAGCCTGGAC
hTRBV10ext	TGTWCTGGTATCGACAAGACC	hTRBV10int	TCCYCCTCACTCTGGAGTC
hTRBV11ext	CGATTTTCTGCAGAGACGC	hTRBV11int	GACTCCACTCTCAAGATCCA
hTRBV12ext	ARGTGACAGARATGGGACAA	hTRBV12int	CYACTCTGARGATCCAGCC
hTRBV13ext	AGCGATAAAGGAAGCATCC	hTRBV13int	CATTCTGAACTGAACATGAGC
hTRBV14ext	CCAACAATCGATTCTTAGCTG	hTRBV14int	ATTCTACTCTGAAGGTGCAGC
hTRBV15ext	AGTGACCCTGAGTTGTTCTC	hTRBV15int	ATAACTTCCAATCCAGGAGG
hTRBV16ext	GTCTTTGATGAAACAGGTATGC	hTRBV16int	CTGTAGCCTTGAGATCCAGG
hTRBV17ext	AGTTGCTGATTTCCTTCCAG	hTRBV17int	CACGCTGAAGATCCATCC
hTRBV18ext	CATAGATGAGTCAGGAATGCC	hTRBV18int	CGATTTTCTGCTGAATTTCC
hTRBV19ext	AGTTGTGAACAGAATTTGAACC	hTRBV19int	TTCCTCTCACTGTGACATCG
hTRBV20ext	AAGTTTCTCATCAACCATGC	hTRBV20int	ACTCTGACAGTGACCAGTGC

hTRBV23ext	GCGATTCTCATCTCAATGC	hTRBV23int	GCAATCCTGTCCTCAGAAC
hTRBV24ext	CCTACGGTTGATCTATTACTCC	hTRBV24int	GATGGATACAGTGTCTCTCGA
hTRBV25ext	ACTACACCTCATCCACTATTCC	hTRBV25int	CAGAGAAGGGAGATCTTTCC
hTRBV2728ext	TGGTATCGACAAGACCCAG	hTRBV2728int	TTCYCCCTGATYCTGGAGTC
hTRBV29ext	TTCTGGTACCGTCAGCAAC	hTRBV29int	TCTGACTGTGAGCAACATGAG
hTRBV30ext	TCCAGCTGCTCTTCTACTCC	hTRBV30int	AGAATCTCTCAGCCTCCAGAC
hTRBCext	TAGAACTGGACTTGACAGCG	hTRBCint	TTCTGATGGCTCAAACACAG
hTRAV1ext	AACTGCACGTACCAGACATC	hTRAV1int	GCACCCACATTTCTKTCTTAC
hTRAV2ext	GATGTGCACCAAGACTCC	hTRAV2int	CACTCTGTGTCCAATGCTTAC
hTRAV3ext	AAGATCAGGTCAACGTTGC	hTRAV3int	ATGCACCTATTCAGTCTCTGG
hTRAV4ext	CTCCATGGACTCATATGAAGG	hTRAV4int	ATTATATCACGTGGTACCAACAG
hTRAV5ext	CTTTTCCTGAGTGTCCGAG	hTRAV5int	TACACAGACAGCTCCTCCAC
hTRAV6ext	CACCCTGACCTGCAACTATAC	hTRAV6int	TGGTACCGACAAGATCCAG
hTRAV7ext	AGCTGCACGTACTCTGTCAG	hTRAV7int	ACAATTTGCAGTGGTACAGG
hTRAV8-1ext	CTCACTGGAGTTGGGATG	hTRAV8-1int	GTCAACACCTTCAGCTTCTC
hTRAV8-3ext	CACTGTCTCTGAAGGAGCC	hTRAV8-3int	TTTGAGGCTGAATTTAAGAGG
hTRAV8-24ext	GCCACCCTGGTTAAAGG	hTRAV8-24int	AGAGTGAAACCTCCTTCCAC
hTRAV8-6ext	GAGCTGAGGTGCAACTACTC	hTRAV8-6int	AACCAAGGACTCCAGCTTC
hTRAV8-7ext	CTCTGGAACTGAAGTGCAAC	hTRAV8-7int	GGAGTTCCTTCTCTCTTCTGG
hTRAV9-1ext	TGGTATGTCCAATATCCTGG	hTRAV9-1int	GAAACCACTTCTTTCCACTTG
hTRAV10ext	CAAGTGGAGCAGAGTCCTC	hTRAV10int	GAAAGAACTGCACTCTTCAATG
hTRAV12-1ext	CARTGTTCCAGAGGGAGC	hTRAV12-1int	AAGATGGAAGGTTTACAGCAC
hTRAV13-1ext	CATCCTTCAACCCTGAGTG	hTRAV13-1int	TCAGACAGTGCCTCAAACTAC
hTRAV13-2ext	CAGCGCCTCAGACTACTTC	hTRAV13-2int	CAGTGAAACATCTCTCTCTGC
hTRAV14ext	AAGATAACTCAAACCCAACCAG	hTRAV14int	AGGCTGTGACTCTGGACTG
hTRAV16ext	AGTGGAGCTGAAGTGCAAC	hTRAV16int	GTCCAGTACTCCAGACAACG
hTRAV17ext	GGAGAAGAGGATCCTCAGG	hTRAV17int	CCACCATGAACTGCAGTTAC
hTRAV18ext	AGAAAACCAGGAGACGGAC	hTRAV18int	CAGGCCAGTCCTATCAAGAG

hTRAV19ext	AGGTAACTCAAGCGCAGAC	hTRAV19int	TGTGACCTTGGACTGTGTG
hTRAV20ext	CACAGTCAGCGGTTTAAGAG	hTRAV20int	TCTGGTATAGGCAAGATCCTG
hTRAV21ext	TTCCTGCAGCTCTGAGTG	hTRAV21int	AACTTGGTTCTCAACTGCAG
hTRAV22ext	GTCCTCCAGACCTGATTCTC	hTRAV22int	CTGACTCTGTGAACAATTTGC
hTRAV23ext	TGCTTATGAGAACACTGCG	hTRAV23int	TGCATTATTGATAGCCATACG
hTRAV24ext	CTCAGTCACTGCATGTTCAG	hTRAV24int	TGCCTTACACTGGTACAGATG
hTRAV25ext	GGACTTCACCACGTACTGC	hTRAV25int	TATAAGCAAAGGCCTGGTG
hTRAV26-1ext	GCAAACCTGCCTTGTAATC	hTRAV26-1int	CGACAGATTCACTCCCAG
hTRAV26-2ext	AGCCAAATTCAATGGAGAG	hTRAV26-2int	TTCACTTGCCTTGTAACCAC
hTRAV27ext	TCAGTTTCTAAGCATCCAAGAG	hTRAV27int	CTCACTGTGTACTGCAACTCC
hTRAV29ext	GCAAGTTAAGCAAAATTCACC	hTRAV29int	CTGCTGAAGGTCCTACATTC
hTRAV30ext	CAACAACCAGTGCAGAGTC	hTRAV30int	AGAAGCATGGTGAAGCAC
hTRAV34ext	AGAACTGGAGCAGAGTCCTC	hTRAV34int	ATCTCACCATAAACTGCACG
hTRAV35ext	GGTCAACAGCTGAATCAGAG	hTRAV35int	ACCTGGCTATGGTACAAGC
hTRAV36ext	GAAGACAAGGTGGTACAAAGC	hTRAV36int	ATCTCTGGTTGTCCACGAG
hTRAV38ext	GCACATATGACACCAGTGAG	hTRAV38int	CAGCAGGCAGATGATTCTC
hTRAV39ext	CTGTTCCTGAGCATGCAG	hTRAV39int	TCAACCACTTCAGACAGACTG
hTRAV40ext	GCATCTGTGACTATGAACTGC	hTRAV40int	GGAGGCGGAAATATTAAAGAC
hTRAV41ext	AATGAAGTGGAGCAGAGTCC	hTRAV41int	TTGTTTATGCTGAGCTCAGG
